# High thermoelectric performance in metal phosphides MP_2_ (M = Co, Rh and Ir): a theoretical prediction from first-principles calculations†

**DOI:** 10.1039/d2ra04175h

**Published:** 2022-08-23

**Authors:** Chung-Jin Kang, Un-Gi Jong, Yun-Hyok Kye, Chol-Jun Yu

**Affiliations:** Chair of Computational Materials Design (CMD), Faculty of Materials Science, Kim Il Sung University Pyongyang PO Box 76 Democratic People's Republic of Korea ug.jong@ryongnamsan.edu.kp cj.yu@ryongnamsan.edu.kp

## Abstract

Although metal phosphides have good electronic properties and high stabilities, they have been overlooked in general as thermoelectrics based on expectation of high thermal conductivity. Here we propose the metal phosphides MP_2_ (M = Co, Rh and Ir) as promising thermoelectrics through first-principles calculations of their thermoelectric properties. By using lattice dynamics calculations within unified theory of thermal transport in crystal and glass, we obtain the lattice thermal conductivities *κ*_l_ of MP_2_ as 0.63, 1.21 and 1.81 W m^−1^ K at 700 K for M = Co, Rh and Ir, respio ectively. Our calculations for crystalline structure, phonon dispersion, Grüneisen parameters and cumulative *κ*_l_ reveal that such low *κ*_l_ originates from strong rattling vibrations of M atoms and lattice anharmonicity, which significantly suppress heat-carrying acoustic phonon modes coupled with low-lying optical modes. Using mBJ exchange–correlation functional, we further calculate the electronic structures and transport properties, which are in good agreement with available experimental data, evaluating the relaxation time of charge carrier within deformation potential theory. We predict ultrahigh thermopower factors as 10.2, 7.1 and 6.4 mW m^−1^ K^2^ at 700 K for M = Co, Rh and Ir, being superior to the conventional thermoelectrics GeTe. Finally, we estimate their thermoelectric performance by computing figure of merit *ZT*, finding that upon *n*-type doping *ZT* can reach ∼1.7 at 700 K specially for CoP_2_. We believe that our work offers a novel materials platform to search for high-performance thermoelectrics using metal phosphides.

## Abstract 2

We investigated the thermoelectric performance of metal phosphides MP_2_ (M = Co, Rh and Ir), such as Seebeck coefficient, electrical conductivity, and lattice and electron thermal conductivity, using the density functional theory calculations.

## Graphical abstract

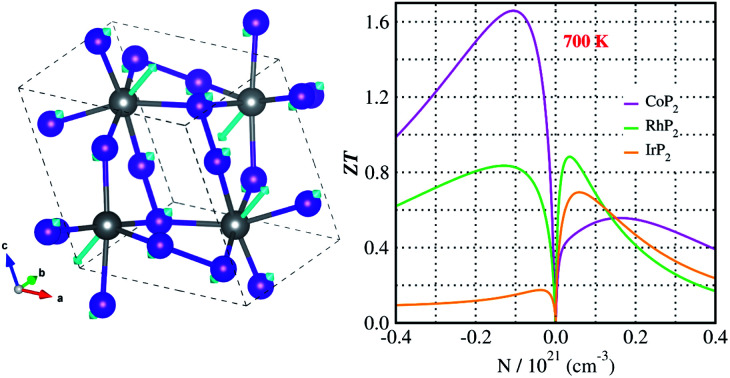

## 1 Introduction

Currently, more than 65% of global energy production is lost as waste heat, being stored in large volumes of warm gases, liquids and solids.^1^ Although no commercially viable technique has yet developed to effectively utilize this waste heat in low energy density, thermoelectric technology has been proved to play an important role in direct conversion of heat into electricity.^2,3^ Like all other heat engines, thermoelectric generators in this technology operate on temperature gradient, Δ*T* = *T*_hot_ − *T*_cold_, and thus the energy conversion efficiency of generator can be enhanced by increasing Δ*T*. Also, the thermoelectric conversion occurs on thermoelectric materials (TEMs), for which a figure of merit is defined as *ZT* = *S*^2^*σT*/*κ*_tot_ with the Seebeck coefficient *S*, electrical conductivity *σ*, and total thermal conductivity *κ*_tot_ as a sum of electronic (*κ*_e_) and lattice vibrational contributions (*κ*_l_).^4^ Given the operational environment of Δ*T*, therefore, the overall efficiency can be enhanced by finding or designing TEMs with high *ZT* values through maximizing both *S* and *σ* while minimizing *κ*_tot_. However, these physical properties is generally interdependent in materials, making it challenging to find innovative TEMs with increasing *ZT* values.^5^

Since the first discovery of the Seebeck phenomena in 1821 and the *ZT* equation in 1911, it had taken several decades to develop the first thermoelectric generator utilizing TEMs.^6^ The most widely used conventional TEMs include group IV and V chalcogenides^7–10^ (*e.g.*, PbTe, Bi_2_Te_3_, Sb_2_Te_3_, SnSe), complex skutterudites^11,12^ and clathrates,^13–15^ and half-Heusler alloys.^16,17^ With these TEMs, the *ZT* value has been considerably increased by advancing materials and synthesis method to increase the thermopower factor *S*^2^*σ* (ref. 18–21) and suppress the lattice thermal conductivity *κ*_l_.^22–26^ For instance, the very high *ZT* value of 2.2–2.6 at 920 K was achieved in PbTe–SrTe binary alloy composites^27^ and SnSe-based solid solutions^28,29^*via* advanced multiple strategies of band engineering, nano-structuring, hierarchical architecturing and non-equilibrium processing. In particular, Zhou *et al.*^30^ recently reported an unusually high *ZT* value approaching ∼3.1 at 783 K, which is the highest record until now, by developing the Na-doped polycrystalline SnSe compounds. To obtain this record, they purified the reactants carefully and removed tin oxide residue from the surface of these compounds, thereby finding the ultralow *κ*_l_ of 0.07 W m^−1^ K together with the high thermopower factor *S*^2^*σ* of 1.2 mW m^−1^ K^2^.

In recent years, unconventional TEMs have been developed and attracted a great deal of research interest. These include halide perovskites,^31,32^ carbon-based semiconductors,^33^ metal complex oxides^34,35^ and metal phosphides,^36,37^ which have several advantages such as high abundance and non-toxicity of the constituent elements, and ease of manufacturing process. Among these TEMs, the most promising material would be a copper phosphide CuP_2_, which combines two desirable properties; the inherent low lattice thermal conductivity and the high thermopower factor through the favorable electronic structure. In fact, Qi *et al.*^36^ demonstrated a much lower *κ*_l_ of 0.6 W m^−1^ K at 300 K in polycrystalline CuP_2_ than the value of ∼1.1 W m^−1^ K for the prototypical TEM SnSe.^29^ Moreover, Odile *et al.*^38^ observed a superior Seebeck coefficient of 692 μ V K^−1^ to the value of ∼190 μ V K^−1^ for SnSe at room temperature. In the meantime, first-principles calculations revealed that the *ZT* value of CuP_2_ can reach over 1.3 at 700 K by optimizing the hole concentration, highlighting a certain possibility of applying metal phosphides to thermoelectric devices.^37^

In spite of some experimental and theoretical studies on CuP_2_, there is a lack of comprehensive insight into thermoelectric performance of metal phosphides. Furthermore, it is expected that replacing Cu atom with other metal atoms in CuP_2_ can enhance the thermoelectric performance. In this work, we perform first-principles calculation to investigate the lattice dynamics, electrical and thermal transport properties of metal phosphides MP_2_ (M = Co, Rh and Ir), the analogues of CuP_2_, which will be used to evaluate their thermoelectric performances. We clarify the underlying fundamental mechanism of enhanced thermoelectric performance so as to design novel TEMs based on metal phosphides.

## 2 Computational methods

The first-principles calculations were carried out using the projector augmented wave (PAW) potential and plane wave method as implemented in the Vienna *Ab initio* Simulation Package (VASP).^39,40^ In the PAW potentials for describing the electrostatic interaction between the ionic cores and valence electrons,^41,42^ the valence electron configurations are Co-3d^8^4s^1^, Rh-4d^8^5s^1^, Ir-5d^8^6s^1^, and P-3s^2^3p^3^. For the exchange–correlation (XC) interaction among the valence electrons, the Perdew–Burke–Ernzerhof (PBE) functional within the generalized gradient approximation (GGA) was employed.^43^ Through explicit convergence test, we set a kinetic energy cutoff for plane wave basis sets to expand Kohn–Sham orbitals as 600 eV and a *Γ*-centered *k*-point mesh for the Brillouin zones (BZ) integration to obtain the electronic density as (8 × 8 × 8) for the unit cell (12 atoms) (see Fig. S1 for convergence test in the ESI†). The crystalline structures were fully optimized both for lattice constants and atomic positions until the magnitude of atomic forces converged to below 10^−3^ eV Å^−1^ with a total energy convergence threshold of 10^−8^ eV. For the lattice dynamics calculations, we built 2 × 2 × 2 supercells (96 atoms), and reduced the cutoff energy as 400 eV and the *k*-point mesh as (4 × 4 × 4), applying the same convergence thresholds. For reliable electronic properties, such as band structure, density of states (DOS) and electronic transport properties, we applied the hybrid XC functional with the modified Becke-Johnson (mBJ) exchange^44^ plus the PBE correlation. This mBJ-PBE functional has been proved to provide well agreed results with the experiment for CuP_2_ in our previous work,^37^ with much less computational efforts compared with HSE06 hybrid functional and explicit many-body GW method. Since in many thermoelectric materials, spin–orbit coupling (SOC) affects the electronic structure and electrical transport, we calculated the electronic band structures and thermopower factors without and with SOC effect.

The electronic transport properties, including the Seebeck coefficient (*S*), electrical conductivity (*σ*) and electron thermal conductivity (*κ*_e_), were evaluated by solving the linearized Boltzmann transport equation (BTE) within the constant relaxation time approximation (CRTA). We implemented the BoltzTrap2 code,^45^ which uses only *k*-dependent quasi-particle energies as input, being approximated by the KS eigenvalues in an efficient procedure. To obtain accurate transport coefficients, therefore, we utilized a denser *k*-point mesh of (24 × 24 × 24) in the first BZ for the band structure calculations. In this work, we assessed the relaxation time of charge carrier by use of the deformation potential theory (DPT), which is a key quantity for determining the electronic transport properties. Within this approach, the relaxation time *τ* is calculated as a function of temperature by considering the electron–phonon interactions mainly contributed from the acoustic phonon modes with the following formula,^37,46^1
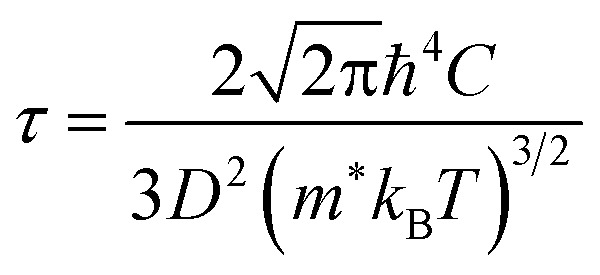
where *C*, *D*, m*, *T*, *ℏ* and *k*_B_ are the elastic constant, deformation potential, effective mass of charge carrier, absolute temperature, reduced Planck constant and Boltzmann constant, respectively. The effective mass defined as m* = ℏ(∂^2^*E*/∂*k*^2^)^−1^ was calculated by using the refined band structures within the single band approximation. The elastic constant *C* was obtained using the density functional perturbation theory (DFPT).^47^ The deformation potential defined as *D* = Δ*E*/(Δ*a*/*a*_0_) was evaluated by calculating the band edge shifts Δ*E* induced by a small change in the lattice constant Δ*a*/*a*_0_.

The harmonic (2nd-order) and cubic (3rd-order) interatomic force constants (IFCs) were calculated by performing the compressive sensing lattice dynamics (CSLD),^48^ as implemented in the ALAMODE code.^49^ In these calculations, the VASP code was used as force calculator to obtain the IFCs with finite displacement method. The 2 × 2 × 2 supercells were used to extract the IFCs. For the harmonic and cubic IFCs, we prepared 15 and 20 different configurations respectively, displaced all the atoms randomly by 0.01 and 0.06 Å from their equilibrium positions, and then computed the atomic forces for each displaced configuration by performing the precise DFT calculations. With the atomic displacement and force data, we extracted the harmonic and cubic IFCs with the CSLD approach, considering all the possible harmonic terms while setting the cut-off radius as 5.1 Å for calculating the cubic IFCs. Using these IFCs, the atomic forces were reproduced within the relative errors less than 4% compared to the DFT-calculated ones. With the harmonic and cubic IFCs, the harmonic phonon dispersions and the lattice transport properties such as phonon group velocity (*v*_g_), phonon lifetime (*τ*_3rd_) and lattice thermal conductivity (*κ*_l_) were calculated by solving the phonon BTE within CRTA. Meanwhile, as Simoncelli *et al.*^50^ lately developed a unified theory of thermal transport in crystals and glasses, the *κ*_l_ was determined by using both the diagonal and off-diagonal elements of phonon velocity operator. When accounting for the diagonal (off-diagonal) contributions to *κ*_l_ only, the unified theory reduced to the Peierls's linearized BTE^51^ (the Allen-Feldman formulation^52^) in the semi-classical limit of a simple crystal (the case of a harmonic glass). In this work, we estimated both the diagonal (*κ*_lP_) and off-diagonal (*κ*_lC_) contributions to the lattice thermal conductivity (*κ*_l_ = *κ*_lP_ + *κ*_lC_) as implemented in the ALAMODE code.

## 3 Results and discussion

### 3.1 Crystalline structure

**Fig. 1:**
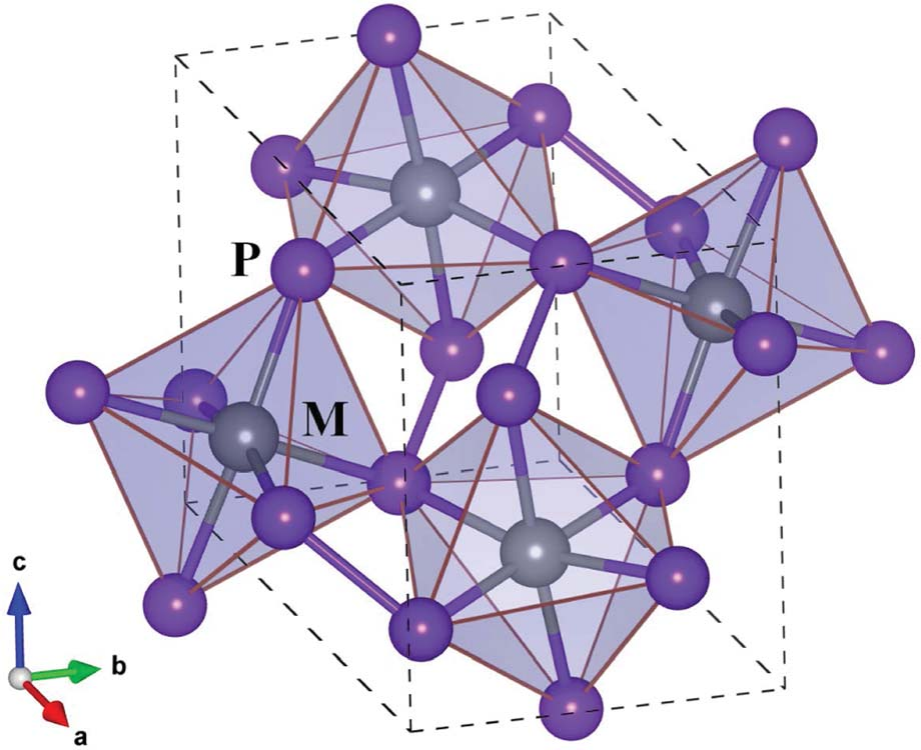
Polyhedral view of crystalline structure of monoclinic MP_2_ (M = Co, Rh, Ir) with a space group of *P*2_1_/*c*. Dashed lines indicate the unit cell. Black- and blue-coloured balls represent M and P atoms, respectively.

Before proceeding to study on the electron and lattice transport properties, we first determined the optimized lattice parameters of metal phosphides MP_2_ (M = Co, Rh and Ir). According to the previous experiments,^53,54^ MP_2_ crystallizes in the monoclinic crystalline phase with a space group of *P*2_1_/*c*. Fig. 1 depicts the unit cell of the monoclinic MP_2_ crystal optimized by using the PBE functional, where metal atoms reside in the corner-sharing octahedra formed by phosphorus atoms. As listed in Table 1, the PBE-calculated lattice constants (angles) were slightly overestimated (underestimated) compared to the previous experiments^53,54^ with relative errors less than 0.9%. On the other hand, the calculated lattice parameters show a distinct variation trend with respect to the choice of metal atom, such that the lattice constant (angle) systematically increases (decreases) as the atomic radius of the metal atom increases, agreeing well with the previous experiments.

**Table 1:** Lattice constants (*a*, *b*, *c*) and angle (*β*), average lattice thermal conductivity (*κ*_l_), band gap (*E*_g_), effective mass (m*), elastic constant (*C*), deformation potential (*D*) and maximum figure of merit (*ZT*) at 700 K for the monoclinic MP_2_ (M = Co, Rh and Ir)

Property	CoP_2_	RhP_2_	IrP_2_
*a*, *b*, *c* (Å)	5.56, 5.54, 5.61	5.78, 5.83, 5.88	5.79, 5.83, 5.89
	5.55, 5.54, 5.61^b^	5.74, 5.79, 5.83^c^	5.74, 5.79, 5.85^c^
*β* (deg)	114.43	112.43	111.29
	114.71^b^	112.91^c^	111.57^c^
*κ*_l_ (W m^−1^ K)	0.63	1.21	1.81
*E*_g_ (eV)	0.44, 0.44^a^	0.45, 0.54^a^	0.73, 0.96^a^
	0.34 ± 0.03^b^		
m*/*m*_h_	0.64^a^	0.68^a^	0.91^a^
*D* (eV)	15.98^a^	18.09^a^	18.97^a^
*C* (GPa)	114.28	122.78^a^	144.31
*n-ZT*	1.68^a^	0.83^a^	0.23^a^
*p-ZT*	0.54^a^	0.92^a^	0.71^a^

a mBJ calculation, otherwise PBE calculation in this work.

b Experimental values from ref. 53.

c Experimental values from ref. 54.

Regarding the crystallographic structure, the local environment around the metal atoms is significantly different in MP_2_ under study compared to CuP_2_, although both of them are in the monoclinic phase with space groups of *P*2_1_/*c*. The difference is that in the monoclinic MP_2_ the local environment is characterized by the corner-sharing MP_6_ octahedra, whereas CuP_2_ has the CuP_4_ tetrahedra distant from each other.^36,37^ Moreover, a layered specific structure, which is consisted of Cu dimer layers and P networks in CuP_2_, is not observed in MP_2_. Such a distinct difference in structure is attributed to the variation of valence electron configurations from Cu-d^10^s^1^ in CuP_2_ to M-d^8^s^1^ (M = Co, Rh, Ir) in MP_2_. On the other hand, the average M–P bond lengths are measured to be 2.25, 2.35 and 2.36 Å, which are much longer than the sum of M and P ionic radii of 1.05, 1.11 and 1.12 Å (ref. 55) for M = Co, Rh and Ir, respectively. Therefore, it is obvious that the metal atoms are located inside the over-sized MP_6_ octahedra, indicating their role as a heavy rattler in MP_2_ crystal.

### 3.2 Lattice dynamics and thermal transport

**Fig. 2:**
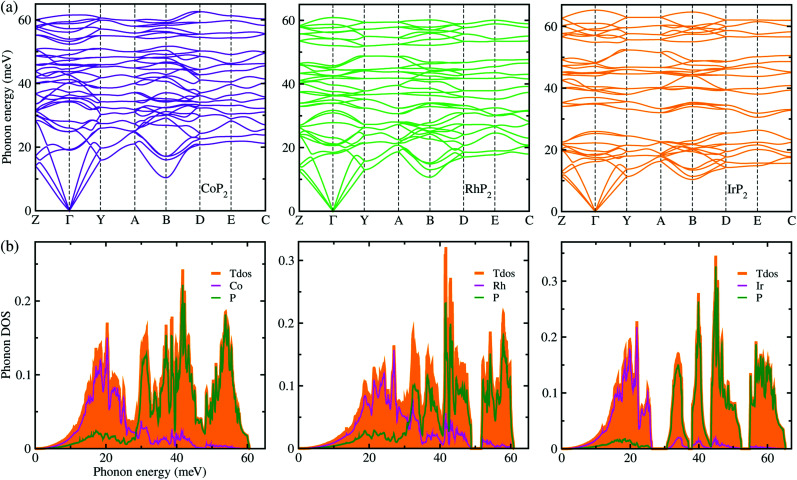
(a) Phonon dispersion curves and (b) atom-resolved phonon DOS for monoclinic MP_2_ (M = Co, Rh and Ir), calculated using 2 × 2 × 2 supercells, harmonic IFCs and PBE functional.

Lattice dynamics and thermal transport properties including *κ*_l_ were studied using the phonon dispersions and cubic IFCs. The phonon dispersions and phonon DOS were firstly calculated using the harmonic IFCs. Fig. 2 shows the calculated phonon dispersion curves and atom-resolved phonon DOS for the monoclinic MP_2_ crystals (M = Co, Rh and Ir). Concerning the accuracy of calculations, we note that the 2 × 2 × 2 supercell calculations give almost identical phonon dispersions with those by 3 × 3 × 3 supercells (see Fig. S2 in the ESI†). No anharmonic phonon modes with imaginary eigenvalues were found in the phonon dispersions, indicating that the monoclinic MP_2_ crystals are dynamically stable at ambient condition as reported in the previous experiments.^53,54^ Meanwhile, the phonon dispersion curves were found to be slightly broadened as going from Co to Rh and to Ir. Moreover, the definite gaps appeared in the high energy regions around 50 meV for RhP_2_ and 30 and 50 meV for IrP_2_, while there is no gap for CoP_2_. Importantly, one can see that the acoustic modes are getting flattened going from the phonon BZ center (*Γ*) to the BZ boundary points. The atomic contributions to the lattice vibrations are found in the atom-resolved phonon DOS as shown in Fig. 2(b). It is found that the metal atoms mainly contribute to the acoustic and low-lying optical modes below ∼25 meV, whereas the P atoms show the dominant contribution to the high energy optical modes.

**Fig. 3:**
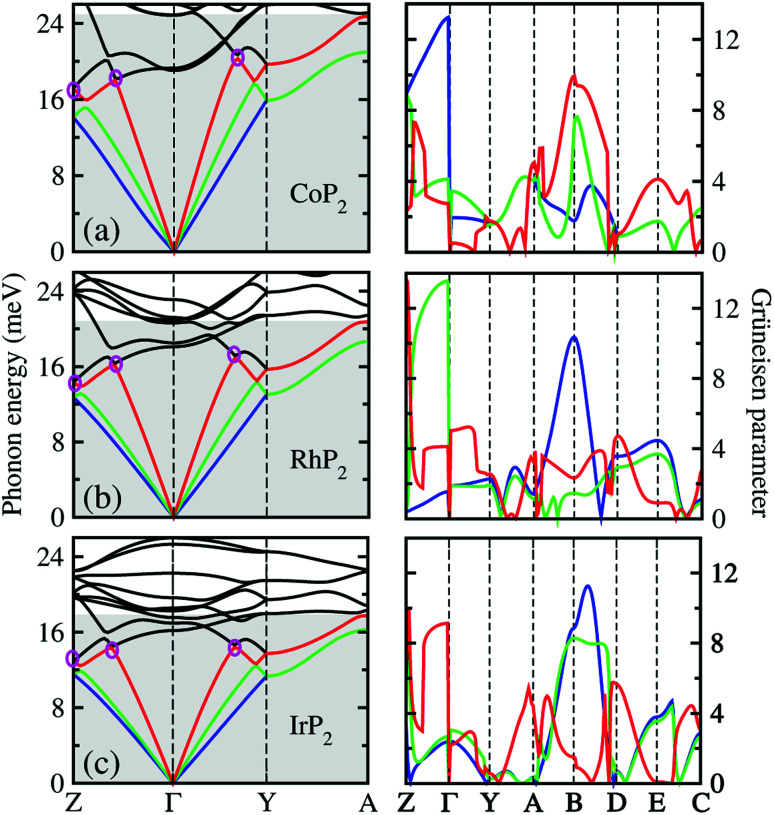
Phonon dispersion curves in acoustic phonon region (gray-coloured region) around the *Γ* point (left panel) and the corresponding Grüneisen parameters (right panel) for (a) CoP_2_, (b) RhP_2_ and (c) IrP_2_. A longitudinal (red) and two transverse acoustic modes (blue and green) are plotted by the colorful solid lines, while the low-lying optical modes by the black solid lines. Magenta-coloured circles indicate the avoided-crossing points between the low-lying optical and the longitudinal acoustic modes.

To get an insightful understanding of the lattice dynamics, we looked deep into the phonon dispersions near the acoustic phonon region. Fig. 3 shows the magnified phonon dispersion curves in the acoustic phonon region with phonon energies ranging from 0 to 26 meV, where a longitudinal (red) and two transverse (blue and green) acoustic branches are plotted as colorful solid lines. The low-lying optical modes were found to be clearly coupled with the acoustic phonon modes, as they appeared in the acoustic region around 20 meV at *Γ* point. Meanwhile, the magenta-coloured circles indicate the avoided-crossing points between the longitudinal acoustic and low-lying optical modes, also confirming the strong optic-acoustic coupling. Moreover, the low-lying optical modes coupled with the acoustic modes were found to be dominated by the metal atom vibrations as shown in Fig. 3(b) for the phonon DOS. Based on these considerations, we conceived that the metal atoms play a certain role in rattling vibrations for the acoustic and low-lying optical phonon modes below ∼26 meV, as expected during the above discussion for the crystalline structures.

We also calculated the Grüneisen parameters corresponding to the acoustic phonon branches shown in Fig. 3, as these parameters are generally accepted as a direct measure of anharmonicity in the lattice vibrations. It was found that the Grüneisen parameters reach the maximum values over 10 along the *Z*–*Γ* line of the phonon BZ with average values of 3.61, 3.31 and 3.18 for M = Co, Rh and Ir, respectively. These are comparable to the average value of 4.1 for SnSe, suggesting strong anharmonicity of the lattice vibrations in the monoclinic MP_2_. On the other hand, as going from M = Ir to Rh and to Co, the average Grüneisen parameter increases gradually, indicating stronger lattice anharmonicity. We note that the rattling vibrations of the metal atoms (discussed below) and lattice anharmonicity will suppress the lattice thermal conductivity in these metal phosphides.

**Fig. 4:**
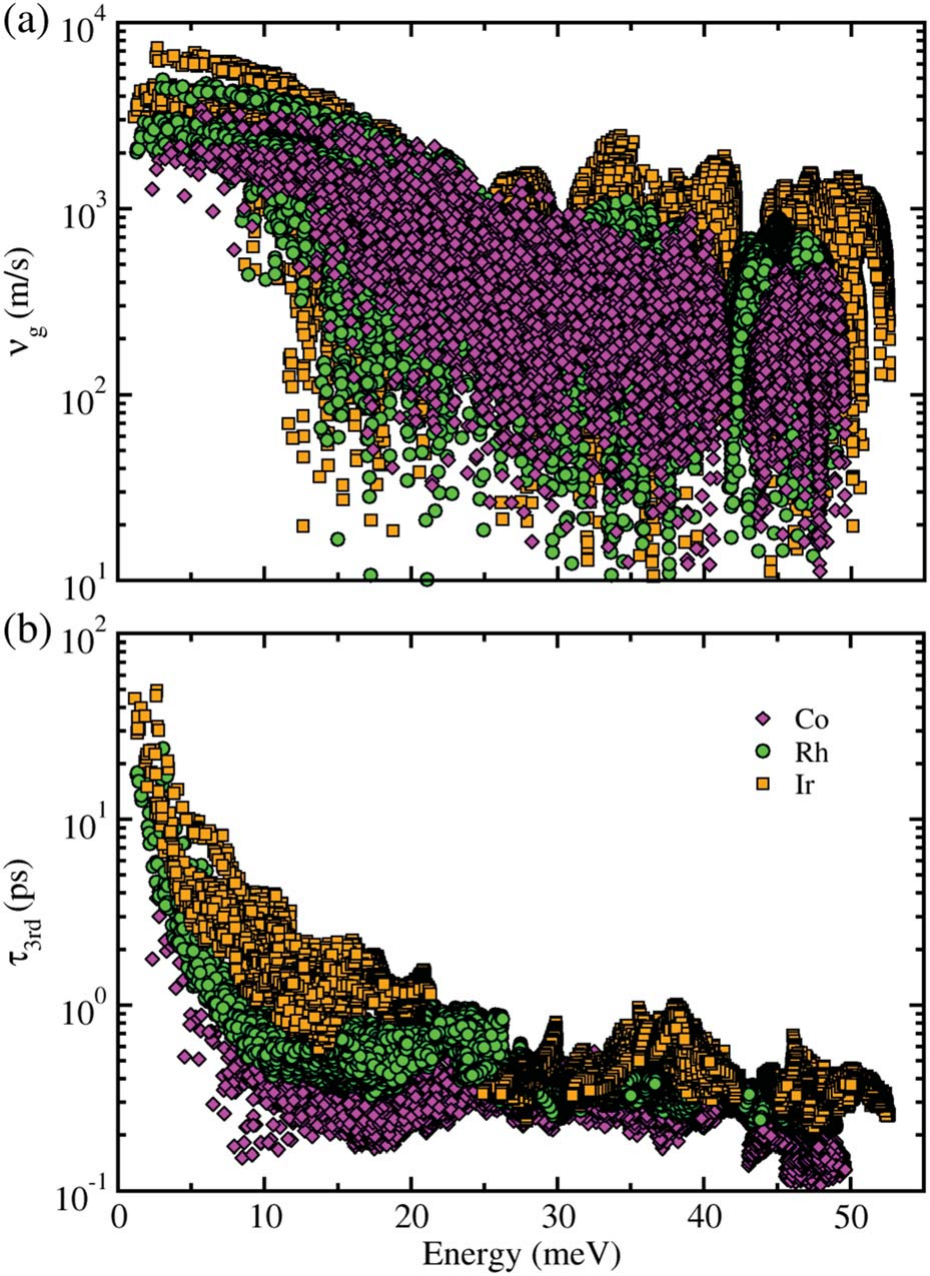
(a) Phonon group velocity *v*_g_ and (b) phonon lifetime *τ*_3rd_ as a function of phonon energy at 700 K for monoclinic MP_2_ (M = Co, Rh, Ir).

Next, within the single-mode CRTA to the phonon BTE, the Peierls's contribution to the lattice thermal conductivity *κ*_lP_ was calculated by summing contributions from the individual phonon modes *λ* as *κ*_lP_ = Σ_*λ*_*C*_V*λ*_|*v*_g*λ*_|^2^*τ*_*λ*_, where *C*_V*λ*_ is the mode-dependent heat capacity, *v*_g*λ*_ the group velocity and *τ*_*λ*_ the phonon lifetime. The heat capacity and phonon group velocity were determined from the phonon dispersions calculated within the harmonic approximation, while the phonon lifetime was calculated by using the 3rd-order IFCs. According to the experimental fact that the metal phosphides such as CuP_2_ and CoP_2_ can be synthesized in well-crystallized form at around 900 K,^36,53^ we investigated the lattice thermal transport and thermoelectric properties at temperatures below 700 K for MP_2_ (M = Co, Rh and Ir). Our calculations revealed that the heat capacity increases rapidly as increasing temperature from 0 to 400 K and slowly after that, and substantially increases as going from M = Co to Rh and to Ir (see Fig. S3 in the ESI†). The phonon group velocity *v*_g_ and phonon lifetime *τ*_3rd_ at 700 K as a logarithmic function of phonon energy were shown in Fig. 4. At the *Γ* point, *v*_g_ was estimated to be about 6500 m s^−1^, which is comparable to the value of CuP_2_ but relatively large compared to those (1000–2000 m s^−1^) of conventional TEMs such as SnSe and PbTe. Meanwhile, *τ*_3rd_ calculated with the 3rd-order IFCs was found to be below 40 ps (see Fig. S4 in the ESI†), which is one order of magnitude smaller than that of SnSe. Furthermore, *τ*_3rd_ was found to rapidly decrease as increasing the phonon energy from 0 to 25 meV. Such rapid drop in *τ*_3rd_ is well coincident with the sharply peaked phonon DOS contributed from the metal atoms, which might be due to the strong phonon scattering by rattling vibrations of the metal atoms as will be discussed below. Finally, it is worth noting that *v*_g_ and *τ*_3rd_ follow the same variation tendency to the heat capacity as varying the species of metal atom from M = Co to Rh and to Ir in the metal phosphides MP_2_ (see Fig. S14 for the average *v*_g_ as a function of temperature in ESI†).

**Fig. 5:**
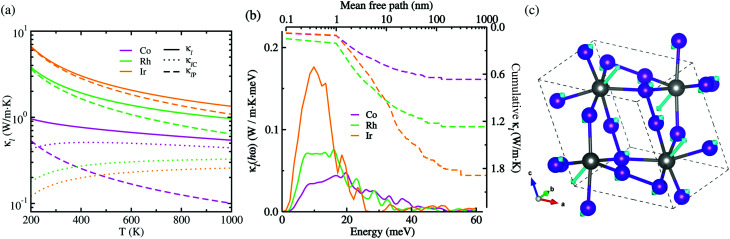
(a) Lattice thermal conductivity *κ*_l_ (solid lines) with Peierls's contribution *κ*_lP_ (dashed lines) and coherent contribution *κ*_lC_ (dotted lines) as increasing temperature *T*, (b) thermal conductivity spectra *κ*_l_(*hω*) as a function of phonon energy (solid lines) and cumulative *κ*_l_ as a function of mean free path (dashed lines) at 700 K, and (c) ball-and-stick view of rattling vibrations of metal atoms corresponding to the lowest optical phonon mode at the *Γ* point of the phonon BZ for the monoclinic MP_2_ (M = Co, Rh, Ir), where the cyan-coloured arrow indicates the atomic displacement vector.

Given *C*_V_, *v*_g_ and *τ*_3rd_ as calculated in this work, we assessed the Peierls's contribution *κ*_lP_ to the lattice thermal conductivity, while the coherent contribution *κ*_lC_ was determined from the off-diagonal elements of the phonon velocity operator (see Fig. S5 for convergence test in the ESI†). Fig. 5(a) shows the total lattice thermal conductivity *κ*_l_ evaluated by summing *κ*_lP_ and *κ*_lC_ (see Fig. S6 for the Cartesian components in the ESI†). In this figure, it was found that at increasing temperature the total lattice thermal conductivity decreases for the three kinds of metal phosphides since the Peierls's contribution decreases as well but the much weaker coherent contribution inceases, as confirmed in the previous work.^50^ At the high-temperature limit, the asymptotic relation of *κ*_lP_ ∝ *T*^−1^ was satisfied for the Peierls's contribution,^51^ which is however in broad disagreement with experiments in most anharmonic and/or complex crystals. When adding the coherent contribution, the total lattice thermal conductivity *κ*_l_ was found to decrease much milder with increasing temperature than the *T*^−1^ tendency. Specifically, at 700 K, the total *κ*_l_ was calculated to be 0.63, 1.21 and 1.81 W m^−1^ K for CoP_2_, RhP_2_ and IrP_2_, respectively, being much lower than or comparable to the value of 1.75 W m^−1^ K for CuP_2_,^36^ which might be due to the rattling vibrations of the metal atoms as depicted in Fig. 5(c). This implies that the metal phosphides can exhibit better thermoelectric performance than CuP_2_. As going from Co to Rh and to Ir, the metal phosphide was found to have larger *κ*_l_ at the given temperature owing to larger *C*_V_, *v*_g_ and *τ*_3rd_. The larger *κ*_l_ is attributed to the weaker lattice anharmonicity and the smaller Grüneisen parameter. On the other hand, the theoretical investigation estimated the amorphous limit of *κ*_l_ to be 1.78 W m^−1^ K at 300 K for CoP_2_,^56^ which is much smaller than our calculation value of ∼8.7 W m^−1^ K for the single crystalline CoP_2_.

Fig. 5(b) presents the thermal conductivity spectra *κ*_l_(*hω*) as a function of phonon energy and the cumulative *κ*_l_ as a function of mean free path (MFP) calculated at 700 K. It can be seen that the low-lying optical phonon modes coupled with the acoustic modes below 25 meV provide dominant contribution to the total *κ*_l_, whereas the high-energy optical modes have negligible contribution. The calculated cumulative *κ*_l_ curves revealed that the maximum length of MFP is about 200 nm and the dominant heat-carrying phonon modes have the mean free pass ranging from 1 to 60 nm for MP_2_. We note, in passing, that nano-structuring can significantly decrease *κ*_l_ of the sample particles with a size smaller than 40 nm in MP_2_, which is more beneficial for enhancing its thermoelectric performance.

### 3.3 Electronic structure

**Fig. 6:**
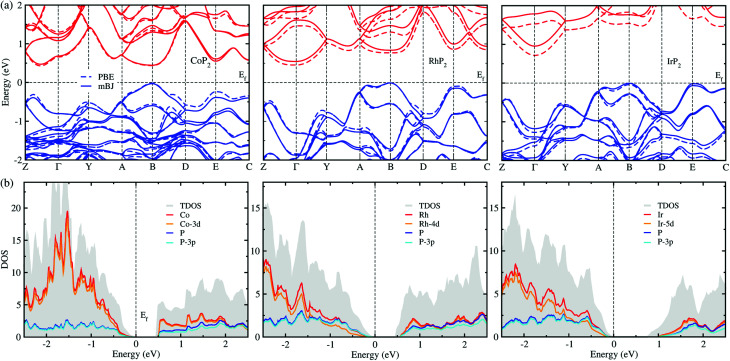
(a) Electronic band structures calculated with the PBE (dashed lines) and mBJ (solid lines) functionals. Conduction and valence bands are represented by blue and red colors, respectively. (b) Total DOS (TDOS) and atom-projected partial DOS obtained with the mBJ functional. The Fermi level *E*_f_ is set to zero and marked by black dashed lines.

The electronic properties calculated in this work includes the band structure and DOS, which will be used to evaluate the electronic transport properties of TEMs. Fig. 6(a) shows the electronic band structure calculated using the mBJ and PBE XC functionals for comparison for the metal phosphides MP_2_. For the case of CoP_2_, the direct transition was found at the *B* point of the electron BZ, whereas for the cases of RhP_2_ and IrP_2_ the indirect transition were found from the valence band maximum (VBM) at the *B* point to the conduction band minimum (CBM) at the *Γ* point. This indicates that CoP_2_ is much more advantageous to creation of charge carriers than RhP_2_ and IrP_2_. For all the three compounds, the band structures are characterized by relative flatness on the top of VBs along the A–B and E–C lines while highly dispersive along the other lines. Moreover, both the VBs and CBs were observed to be doubly degenerated along the Y–A and D–E–C lines. Such band characteristics are revealed irrespective of the choice of XC functional, leading to high thermopower factor for the metal phosphides like the semiconductor-based TEMs. Fig. 6(b) displays the mBJ-calculated total and atom-projected partial DOS. It was found that both the CBs and VBs are characterized by strong p–d hybridization between d states of metal atom and 3p states of phosphorus atom.

For comparison between the mBJ and PBE calculations, we found that the mBJ calculations slightly push up the conduction bands, thus widening the band gap *E*_g_, compared with the PBE calculations. This effect is getting more pronounced as going from Co to Rh and to Ir. As listed in Table 1, the mBJ functional yielded larger band gaps of 0.44, 0.54 and 0.96 eV than the values of 0.44, 0.45 and 0.73 eV by the PBE functional for Co, Rh and Ir phosphides, respectively. We note that the band gap of 0.44 eV calculated in this work with the PBE and mBJ functionals are in excellent agreement with the previous PBE-calculation value of 0.44 eV for CoP_2_.^56^ According to the previous work,^17^ such narrow band gaps can give rise to the large *σ* and *S*, therefore enhancing the thermopower factor *S*^2^*σ*. The mBJ functional is generally able to give more reliable band structures in comparison with experiments, as confirmed in our previous works for CuP_2_ and CsAg_5_Te_3_.^37,46^ For the case of CoP_2_, both the mBJ and PBE functionals give the same band gap of 0.44 eV in reasonable agreement with the experimental value of 0.34 ± 0.03 eV which was determined as a slope of temperature-dependent specific electrical resistivity for CoP_2_.^53^ As going from M = Co to Rh and to Ir, the band gap increases clearly, indicating that CoP_2_ is more favorable for generating charge carriers due to the lower direct band gap compared with the higher indirect band gaps of RhP_2_ and IrP_2_. In addition, we calculated DOS of CoP_2_ by employing the HSE06 hybrid functional and LDA + *U* approach with *U* = 4 eV for Co-3d states, yielding the band gaps of 0.82 and 0.59 eV, respectively (see Fig. S7 and S8 in the ESI†). These values are far larger than the experimental value. Therefore, we used only the mBJ functional to estimate the electronic transport properties as will be discussed below.

### 3.4 Thermoelectric performance

At the final step, we calculated the electrical conductivity *σ*, Seebeck coefficient *S*, thermopower factor *S*^2^*σ* and figure of merit *ZT* for thermoelectric performance. Firstly, the relaxation time *τ* of charge carrier was estimated as a function of temperature by using eqn (1). To do this, we calculated the deformation potential *D* representing the electron-acoustic phonon interaction and the effective mass m* of charge carriers from the calculated band structures (see Fig. S9 for *D* in the ESI†). The elastic constant *C* was calculated by applying the DFPT method. Table 1 lists the calculated values for the metal phosphides MP_2_ (M = Co, Rh and Ir). It was found that *D*, m* and *C* increase gradually as going from Co to Rh and to Ir. Given these quantities of *D*, m* and *C*, we could assess the relaxation time *τ* as a function of temperature (see Fig. S4 in the ESI†), demonstrating that *τ* decreases as increasing temperature and also going from Co to Rh and to Ir at the given temperature.

**Fig. 7:**
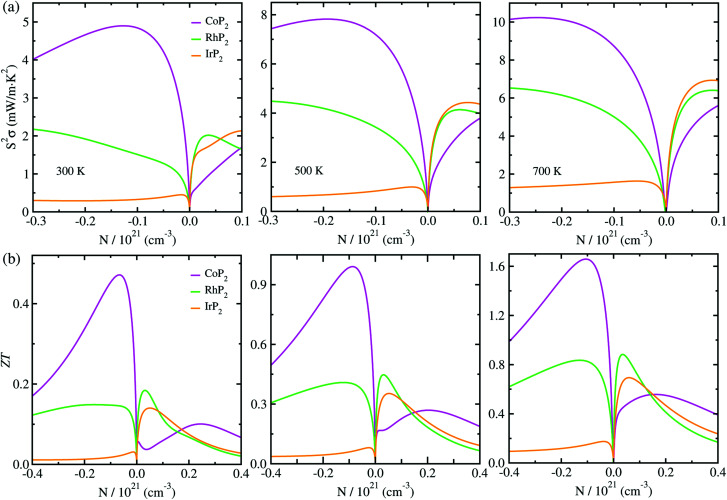
(a) Thermopower factor *S*^2^*σ* and (b) figure of merit *ZT* as functions of carrier concentration N, calculated with the mBJ functional at *T* = 300, 500 and 700 K for the monoclinic MP_2_ (M = Co, Rh and Ir).

Then, we estimated the thermopower factor *S*^2^*σ* as a function of carrier concentration at temperatures of 300, 500 and 700 K, as shown in Fig. 7(a). It was found that by optimizing the carrier concentration the metal phosphides can exhibit the maximum *S*^2^*σ* values of 10.2, 7.1 and 6.4 mW m^−1^ K^2^ at 700 K for M = Co, Rh and Ir, respectively. These estimated values are larger than the values of 6.4 and 3.3 mW m^−1^ K^2^ at 700 K for CuP_2_ (ref. 37) and conventional TEM GeTe,^8^ thereby leading to higher thermoelectric performance for MP_2_. As going from M = Co to Rh and Ir, meanwhile, the maximum *S*^2^*σ* value was found to decrease at the given temperature due to the decrease of the relaxation time. For the case of IrP_2_, the *n*-type power factor is shown to decrease slowly according to the increment of electron concentration, indicating that the electron–electron scattering is so weak that it has small influence on the electronic transport properties.

Finally, we determined the figure of merit *ZT* as a function of carrier concentration at temperatures of 300, 500 and 700 K, as shown in Fig. 7(b) (see Fig. S10 for convergence test in the ESI†). It was observed that *ZT* exhibits the similar variation tendency to *S*^2^*σ* for selection of metal atom; the maximum *ZT* value at the given temperature decreases as going from M = Co to Rh to Ir, because *S*^2^*σ* decreases while *κ*_l_ increases according the change of metal atom in MP_2_. Our calculations revealed that the *n*-type doping can enhance the thermoelectric performance than the *p*-type doping at the given temperature for CoP_2_, but *vice versa* for RhP_2_ and IrP_2_. Such inversion in the favorable carrier type is ascribed to the inverse behavior of electronic band structures near the Fermi level for CoP_2_*versus* RhP_2_ and IrP_2_. As shown in Fig. 6(a), some conduction (valence) states appear at *E*, *C* points and along *Z*–*Γ* (A–B) line in the CBs (VBs) with almost the same eigenvalues as CBM (VBM) at *B* point for CoP_2_ (RhP_2_ and IrP_2_) but not for RhP_2_ and IrP_2_ (CoP_2_). These conduction and valence states near the Fermi level can play a positive role in transporting the electrons and holes, respectively. As increasing temperature, the maximum *ZT* was found to be remarkably enhanced for all the metal phosphides.

For CoP_2_, in particular, the *n*-type *ZT* at 700 K reaches about 1.7 at a carrier concentration of about 9 × 10^19^ cm^−3^, which is clearly higher compared to its Cu-based counterpart CuP_2_. As shown in Table 1, the maximum *ZT* values upon *n*-type doping at 700 K were determined to be 0.87 and 0.23 for RhP_2_ and IrP_2_, respectively. Meanwhile, the maximum *p-ZT* values at 700 K are 0.54, 0.92 and 0.71 for CoP_2_, RhP_2_ and IrP_2_, respectively. Especially, the metal phosphides CoP_2_ and RhP_2_ show the high *ZT* values for both *n*- and *p*-type dopings at 700 K, and therefore, the combination of *n*-type CoP_2_ and *p*-type RhP_2_ (or IrP_2_), *i.e.*, *n*-CoP_2_/*p*-RhP_2_ (or *n*-CoP_2_/*p*-IrP_2_), will provide a substantial improvement of thermoelectric performance over the pure CoP_2_ and RhP_2_ (or IrP_2_). Our PBE-calculation of *p-ZT* (0.57) at 700 K is smaller than the previous calculation (0.93) for CoP_2_ by Pohls *et al.*^56^ in which the authors employed the PBE functional and the amorphous limit of *κ*_l_ for calculating the *ZT*. In order to validate our calculations, we checked convergence of the figure of merit *ZT* according to *k*-point mesh and the choice of XC functional (see Fig. S10 and S11 in the ESI†). Meanwhile, we presented the electronic band structures and thermopower factors calculated with considering the SOC effect (see Fig. S12 and S13 in the ESI†), finding that the SOC effect has negligible impact for the metal phosphides MP_2_. It was confirmed that the numerical error of our calculations is within the reasonable magnitude below 0.2. From our calculations, it can be concluded that the metal phosphide CoP_2_ in the monoclinic phase is the most preferable candidate for high performance TEM, while RhP_2_ and IrP_2_ should not be precluded, and the thermoelectric performance can be enhanced by optimizing the carrier concentration.

## 4 Conclusions

In this work, we have performed first-principles calculations to predict the thermoelectric properties of metal phosphides MP_2_ (M = Co, Rh and Ir) in monoclinic phase. Lattice dynamics calculations revealed that these metal phosphides have remarkably low lattice thermal conductivities of 0.63, 1.21 and 1.81 W m^−1^ K at 700 K for M = Co, Rh and Ir, respectively. Through the scrutiny of crystalline structure, it was found that such low *κ*_l_ is most likely attributed to the atomic rattling vibrations of the metal atoms, which strongly scatter the low-lying optical phonon modes coupled with the heat-carrying acoustic modes. Such findings were further confirmed by the analysis of phonon DOS, avoided-crossing behavior of phonon dispersion curves, Grüneisen parameters and cumulative *κ*_l_. By the use of the mBJ XC functional, we calculated the electronic band structures with the band gaps, revealing that CoP_2_ has a direct band gap of 0.44 eV while RhP_2_ and IrP_2_ have indirect larger band gaps of 0.54 and 0.96 eV. These compounds were found to have high thermopower factors of 10.2, 7.1 and 6.4 mW m^−1^ K^2^ at 700 K for M = Co, Rh and Ir, being superior to the conventional thermoelectrics of GeTe. The maximum figures of merit *ZT* were found to reach relatively high values of 1.68, 0.87 and 0.71 at 700 K for M = Co, Rh and Ir by optimizing the carrier concentrations, concluding that CoP_2_ is the most preferable for TEM while RhP_2_ and IrP_2_ should not be precluded. Our work offers a novel materials platform to search for high-performance thermoelectrics from metal phosphides.

## Author contributions

Chung-Jin Kang and Un-Gi Jong developed the original project, performed the calculations and drafted the first manuscript. Yun-Hyok Kye assisted with the DFT calculations and the post-processing of calculation results, and contributed to useful discussions. Chol-Jun Yu supervised the work. All authors reviewed the manuscript.

## Conflicts of interest

There are no conflicts to declare.

## Supplementary Material

RA-012-D2RA04175H-s001
