# Supplementary material for: High thermoelectric performance in metal phosphides MP2 (M = Co, Rh and Ir): a theoretical prediction from first-principles calculations
Source: RSC Adv. 2022 Aug 23;12(37):23829–38. doi: 10.1039/d2ra04175h (PMC9396636; doi:10.1039/d2ra04175h)
Supplement: RA-012-D2RA04175H-s001 [file RA-012-D2RA04175H-s001.pdf]

Electronic Supplementary Material (ESI) for RSC Advances.
This journal is © The Royal Society of Chemistry 2022

Electronic Supplementary Information – High thermoelectric performance in metal phosphides MP_2 (M = Co, Rh and Ir): A theoretical prediction from first-principles calculations

Chung-Jin Kang[a], Un-Gi Jong[a*], Yun-Hyok Kye[a] and Chol-Jun Yu[a†]

[a] *Chair of Computational Materials Design (CMD), Faculty of Materials Science, Kim Il Sung University, Pyongyang, PO Box 76, Democratic People's Republic of Korea*

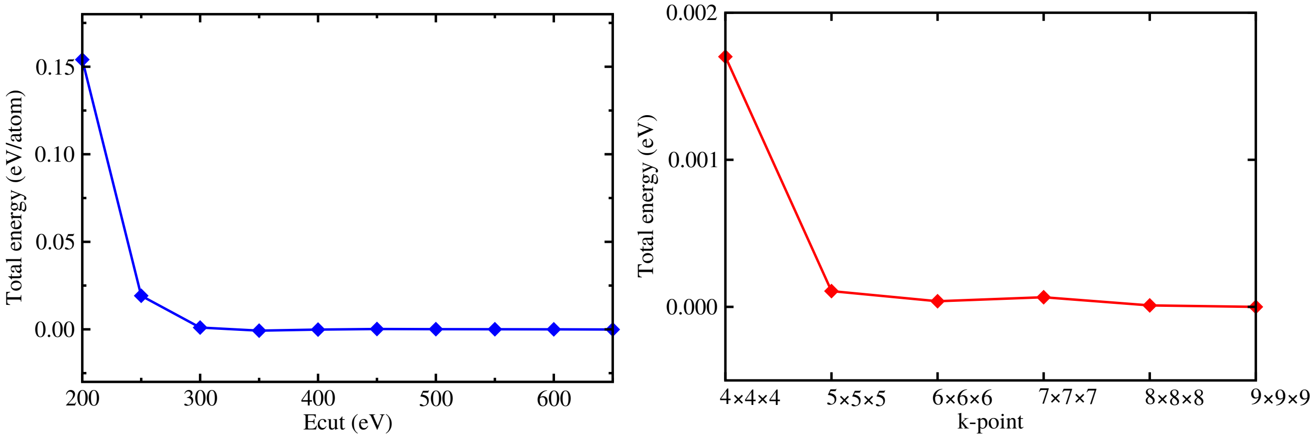

Figure S1. Total energy convergence test as varying the kinetic cutoff energy with a fixed *k*-point mesh of (8×8×8) (left panel) and the *k*-point mesh with a fixed cutoff energy of 600 eV (right panel) for the CoP_2 unit cell.

*Un-Gi Jong, Email: ug.jong@ryongnamsan.edu.kp
†Chol-Jun Yu, Email: cj.yu@ryongnamsan.edu.kp

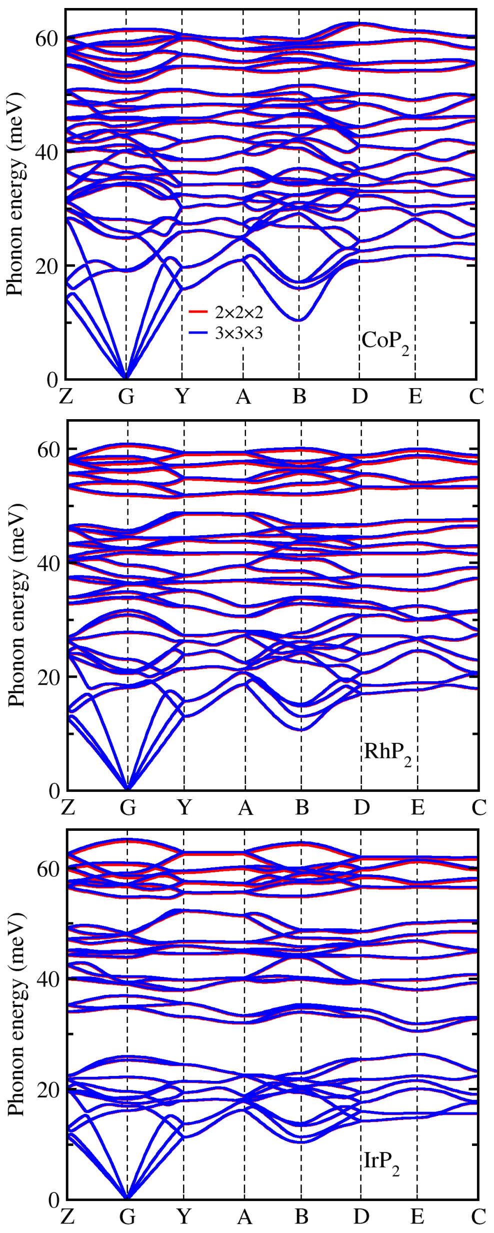

Figure S2. Comparison of the phonon dispersion curves calculated using 2×2×2 (red lines) and 3×3×3 (blue lines) supercells for the monoclinic MP_2 (M = Co, Rh, and Ir).

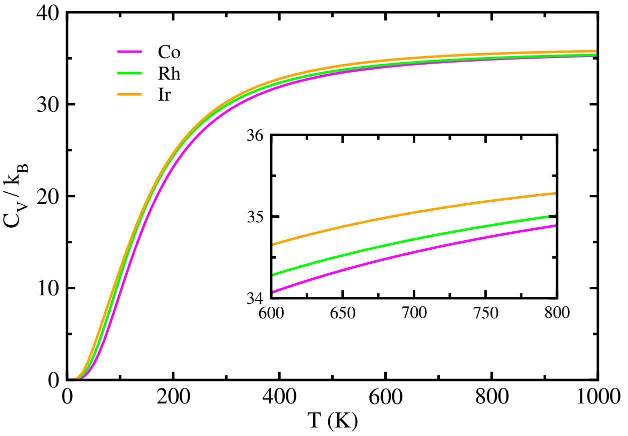

Figure S3. Heat capacity C_V as a function of temperature in MP_2 (M = Co, Rh, Ir).

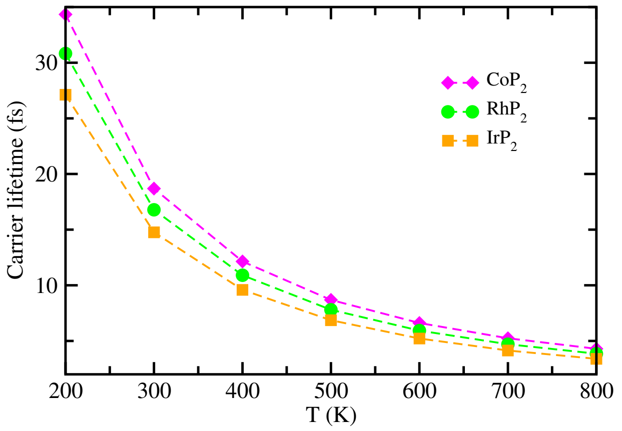

Figure S4. Carrier lifetime calculated as a function of temperature within the deformation potential theory for the MP_2 (M = Co, Rh, and Ir).

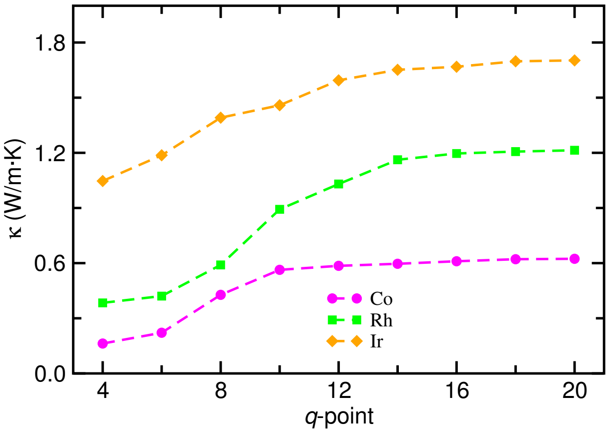

Figure S5. Convergence tests for the lattice thermal conductivity of MP_2 (M = Co, Rh, and Ir) according to the size of q-point mesh in the phonon BZ.

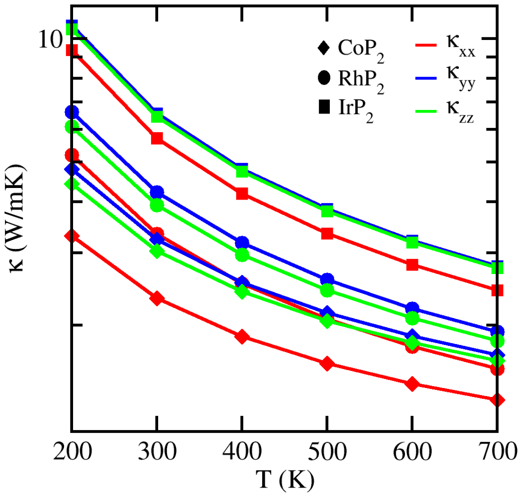

Figure S6. Total lattice thermal conductivity ($\kappa_l = \kappa_{l\mathbf{P}} + \kappa_{l\mathbf{C}}$) as functions of temperature along the x, y, and z-axis for the monoclinic MP_2 (M = Co, Rh, and Ir).

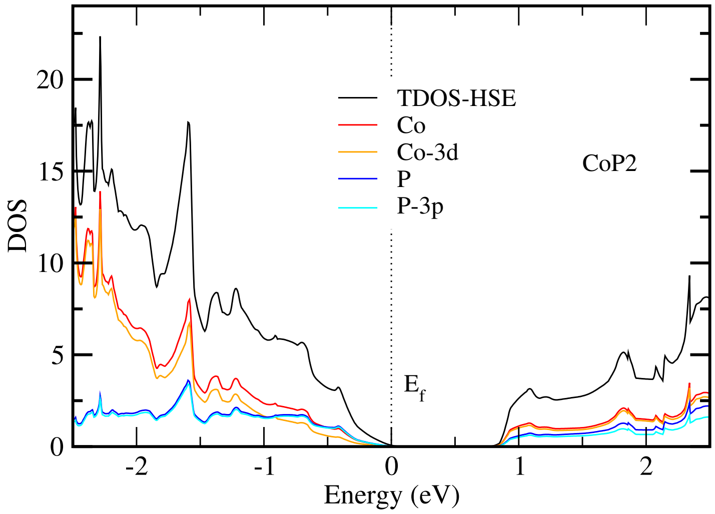

Figure S7. Atom-projected electron density of states calculated by the HSE06 hybrid functional for CoP_2.

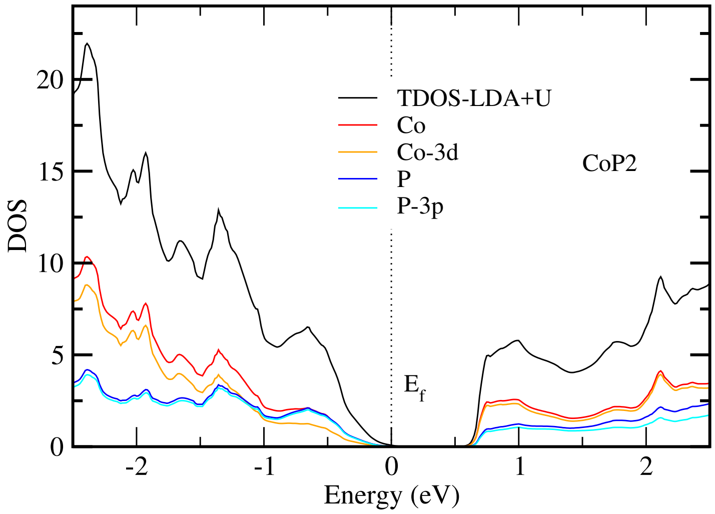

Figure S8. Atom-projected electron density of states calculated by LDA+U approach with U = 4 eV for CoP_2.

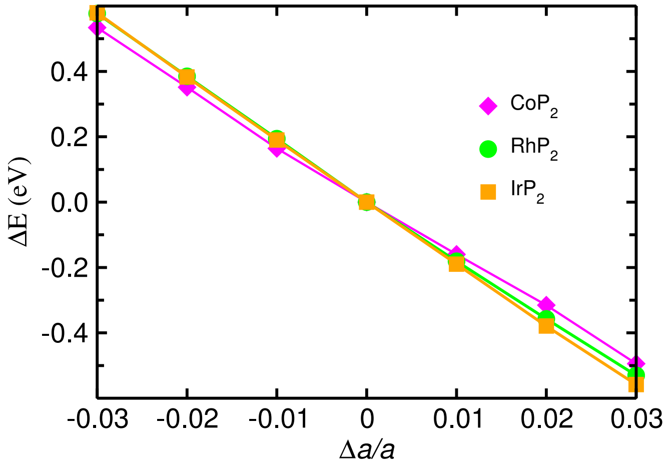

Figure S9. Deformation potential D calculated with mBJ functional for the MP_2 (M = Co, Rh, and Ir).

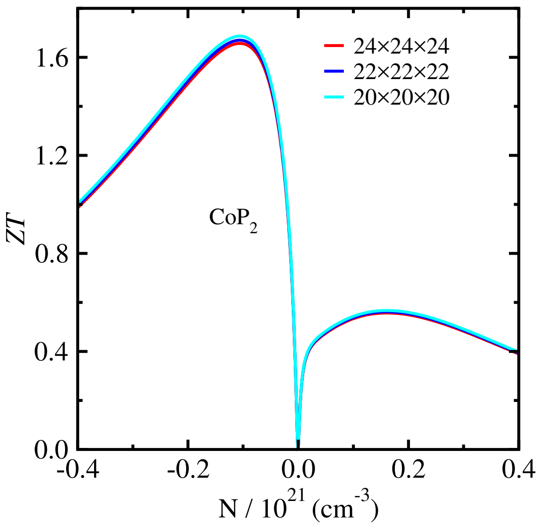

Figure S10. Figure of merit *ZT* calculated at 700 K using different *k*-point meshes for CoP_2.

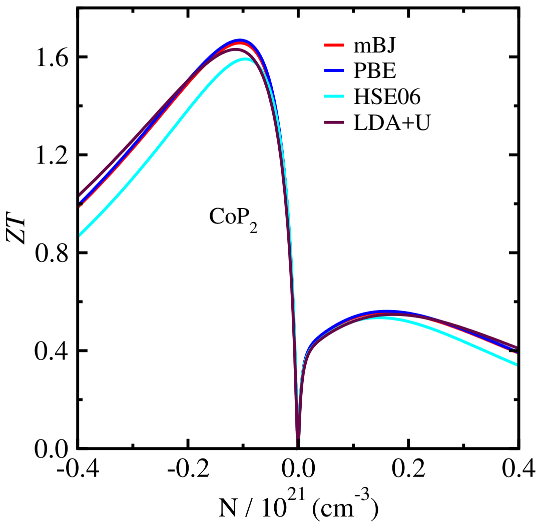

Figure S11. Figure of merit *ZT* calculated at 700 K using different exchange-correlation functionals including PBE, mBJ and HSE06, and using LDA+U approach for CoP_2.

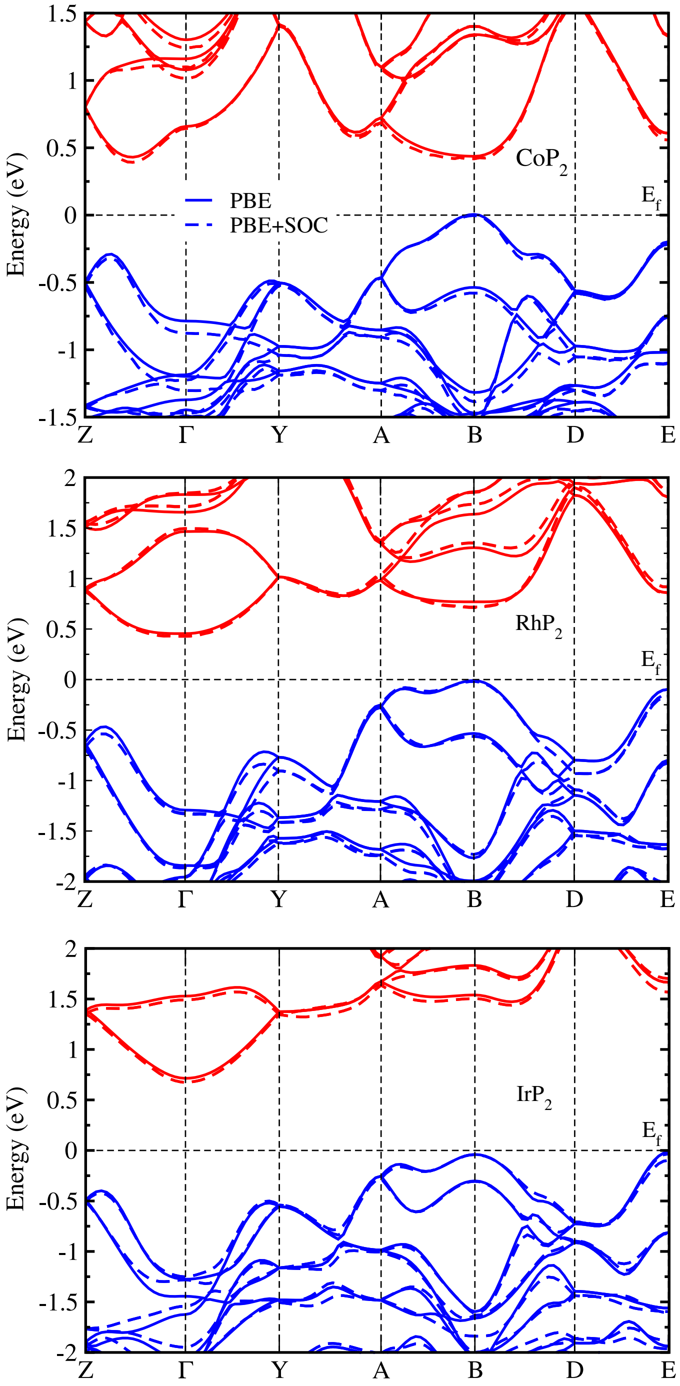

Figure S12. Electronic band structures calculated by using the PBE functional with (dotted) and without (solid) the spin-orbit coupling effect for MP_2 (M = Co, Rh and Ir).

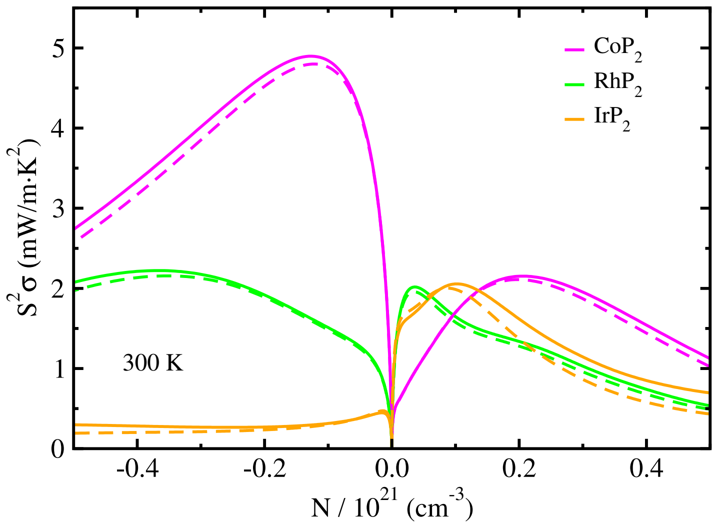

Figure S13. Thermopower factor $S^2\sigma$ calculated at 300 K by using the PBE functional with (dotted) and without (solid) the spin-orbit coupling effect for MP_2 (M = Co, Rh and Ir).

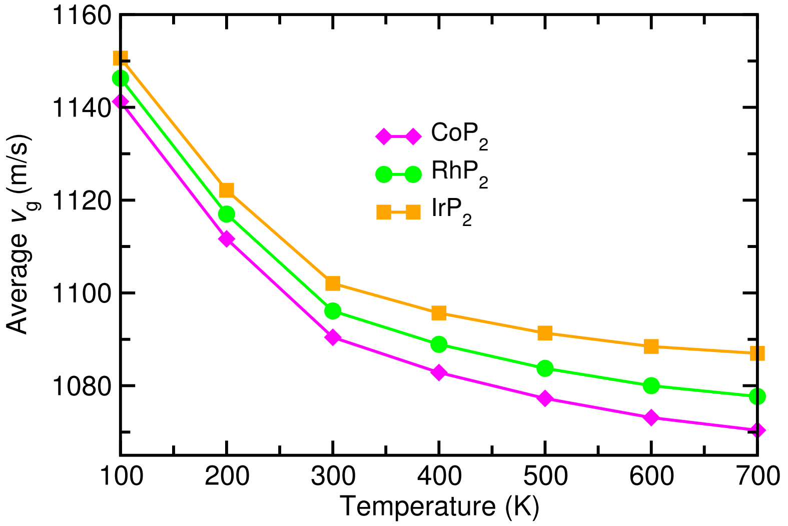

Figure S14. Average phonon group velocity as a function of temperature for MP_2 (M = Co, Rh and Ir).
